# First Efficacy Results of Capecitabine with Anthracycline- and Taxane-Based Adjuvant Therapy in High-Risk Early Breast Cancer: A Meta-Analysis

**DOI:** 10.1371/journal.pone.0032474

**Published:** 2012-03-02

**Authors:** Yiwei Jiang, Wenjin Yin, Liheng Zhou, Tingting Yan, Qiong Zhou, Yueyao Du, Zhenzhou Shen, Zhimin Shao, Jinsong Lu

**Affiliations:** 1 Department of Oncology, Shanghai Medical College, Fudan University, Shanghai, People's Republic of China; 2 Department of Breast Surgery, Fudan University Shanghai Cancer Center, Shanghai, People's Republic of China; University of Kentucky College of Medicine, United States of America

## Abstract

**Background:**

Capecitabine is effective and indicated for the salvage treatment of metastatic breast cancer. Therefore, it is essential to evaluate the efficacy of capecitabine in the adjuvant setting. There have been two large randomized studies to determine whether patients with high-risk early breast cancer benefit from the addition of capecitabine to standard chemotherapy, but they have yielded inconsistent results. We first undertook a meta-analysis to evaluate the efficacy of the addition of capecitabine over standard treatment.

**Methods:**

PubMed, EBSCO, Web of Science, conference proceedings and key trials were searched from 1998 to 2011. The hazard ratio (HR) was used to evaluate the efficacy of a taxane-anthracycline regimen and a taxane-anthracycline-capecitabine regimen in early breast cancer. All of the data from each study use either fixed-effects or random-effects by Stata.

**Findings:**

We found significant improvement in the additional capecitabine arm versus control in disease-free survival (DFS) (HR = 0.83, 95% CI: 0.71–0.98, P = 0.027), overall survival (OS) (HR = 0.71, 95% CI: 0.57–0.88, P = 0.002), distant recurrence (HR = 0.79, 95% CI: 0.66–0.94, P = 0.008) and the death from breast cancer only (HR = 0.65, 95% CI: 0.51–0.83, P = 0.001). Meanwhile, the subgroup analysis revealed that capecitabine improved the DFS in triple negative (HR = 0.71, 95% CI: 0.53–0.96, P = 0.028), hormone receptor negative (HR = 0.73, CI: 0.56–0.94, P = 0.017) and HER2 negative (HR = 0.81, CI: 0.67–0.98, P = 0.034) patients.

**Conclusion:**

Due to the synergistic effect of taxane and capecitabine, taxane-anthracycline-capecitabine regimen may effectively improve the efficacy in the adjuvant setting and may be a novel generation of adjuvant chemotherapy regimen. The results of the current meta-analysis support this hypothesis and indicate that taxane-based regimen with capecitabine may be an effective, convenient, and well tolerated regimen in patients with early breast cancer.

## Introduction

The key milestones for breast cancer treatment were endocrine therapy in the 1960s and polychemotherapy in the 1970s. In recent decades, the development of effective targeted therapies has been another significant milestone in breast cancer treatment [1]. As an adjuvant chemotherapy regimen, cyclophosphamide, methotrexate and 5-fluorouracil (CMF) combination chemotherapy has achieved similar excellent efficacy as in the salvage setting [2]. Anthracyclines appeared in 1972, followed by taxanes in the 1990s. Anthracycline- and taxane-based polychemotherapy regimens have achieved better efficacy than any previous chemotherapy regimen, so they have long been recommended as standard adjuvant regimens in the main breast cancer treatment guidelines, such as the National Comprehensive Cancer Network (NCCN) guidelines and St Gallen Consensus. Based on data from large and well-controlled clinical studies, gemcitabine, vinorelbine and capecitabine have also entered clinical care as salvage treatment for metastatic breast cancer. Unfortunately, some chemotherapy drugs such as vinorelbine and gemcitabine have failed to show superior efficacy in the adjuvant treatment, as the Finland Herceptin Trial and tAnGo trial showed [3], [4].

Capecitabine is a fluoropyrimidine carbamate, which is supplied for oral administration as a systemic prodrug of 5′-deoxy-5-fluorouridine (5′-DFUR). 5′-DFUR is converted to 5-fluorouracil by sequential enzyme activity. The enzyme responsible for the final step is thymidine phosphorylase (TP), which is overexpressed in breast cancer [5]. Some cytotoxic drugs such as docetaxel show synergistic effects with capecitabine, because the former can increase the TP level in tumors [6]. Furthermore, capecitabine combined with docetaxel has improved time to progression and overall survival (OS) in some pivotal phase III trials [7]. For this reason, the NCCN Breast Cancer Guidelines Committee decided to incorporate docetaxel/capecitabine as a preferred combined chemotherapy regimen for recurrent or metastatic breast cancer [8].

Capecitabine is effective and indicated for the salvage treatment of metastatic breast cancer. Therefore, it is essential to evaluate the efficacy of capecitabine-containing regimens in the adjuvant setting. To date, there have been two large randomized, open-label, multicenter phase III studies to determine whether patients with high-risk early breast cancer benefit from the addition of capecitabine to standard chemotherapy, but they have yielded inconsistent results. The Finxx trial suggested that addition of capecitabine to a taxane–anthracycline regimen did not significantly improve recurrence-free survival (HR = 0.79, 95% CI: 0.60–1.04, P = 0.087) or OS (HR = 0.73, 95% CI: 0.52–1.04, P = 0.08) as compared to the taxane–anthracycline regimen. The USON 01062 trial found significant improvement in OS (HR = 0.68, 95% CI: 0.51–0.92, P = 0.011) rather than in disease-free survival (DFS) (HR = 0.84, 95% CI: 0.67–1.05, P = 0.125) in favor of capecitabine combined with a taxane–anthracycline regimen [9], [10], [11].

For the above reasons, we sought to undertake a meta-analysis to evaluate the efficacy of capecitabine when combined with standard treatment in the adjuvant setting for early breast cancer. We compared the outcomes of OS, DFS, local recurrence, distant recurrence and breast-cancer-specific survival because these are the main endpoints used in clinical trials. Besides, we performed subgroup analyses according to hormone receptor and HER2 status as well as triple negativity.

## Methods

### Identification of randomized studies

Two investigators (YW Jiang and WJ Yin) independently obtained relevant English language articles through searches of PubMed, EBSCO and Web of Science databases, conference proceedings (American Society of Clinical Oncology, San Antonio Breast Cancer Symposium and European Society for Medical Oncology) and scanned the reference lists of key trials and review articles from 1998 (based on the first reported trial of capecitabine efficacy in humans) to the end of November 2011. PubMed, EBSCO and Web of Science databases were searched using terms ‘capecitabine’, ‘Xeloda’, and the exploded MeSH term ‘breast neoplasms’. We searched conference proceedings through online websites at www.asco.org, www.esmo.org and www.sabcs.org. We included randomized, open-label, phase III trials in early breast cancer. We excluded trials if they compared the efficacy of capecitabine-based regimens in the salvage and neoadjuvant setting, as well as those to test the efficacy of single capecitabine in the adjuvant setting.

### Study endpoints

In this meta-analysis, the primary outcome was DFS, defined as time from randomization to the first occurrence of disease progression or death from any cause without documentation of a cancer-related event. Secondary outcomes included overall survival (death from any cause), time to distant recurrence, breast-cancer-specific survival (death from breast cancer) and time to death from other causes.

### Data extraction

From each eligible trial, two independent reviewers (YW Jiang and WJ Yin) extracted data, including authors' names, journal, year of publication, trial design, patient eligibility, baseline patient characteristics, dosing regimens, duration of follow-up, and treatment changes due to toxicity. For trials that compared different types of treatment, we derived the number of patients with any recurrence or any death and the total number of patients in each treatment arm. We also derived HRs and 95% CIs for the outcomes OS and DFS, or evaluated the logarithm of the HR for death. All of the outcomes were based on the intention-to-treat (ITT) analysis. If the trial results were reported in multiple publications, we extracted the most recently reported endpoints.

### Statistical analysis

HR was used to evaluate the efficacy of a taxane–anthracycline regimen and a taxane–anthracycline–capecitabine regimen in early breast cancer. For each study, the between-study heterogeneity was assessed by the χ^2^ based Q statistics and I^2^ test. Heterogeneity was considered as either P<0.50 or I^2^>50%. All of the data from each study used either fixed effects (Mantel–Haenszel's method) or random-effects (DerSimonian and Laird's method) models according to the heterogeneity result. If there was no between-study heterogeneity, the two methods provided similar results. Funnel plots and Begger's test were used to test the possible publication bias. Sensitivity analyses were performed to evaluate the influence of individual studies on the summary effect. In the subgroup analysis, statistical analysis was performed in different hormone receptor status, HER2 status and triple negative status. All of the analyses were performed by Stata 10.0 software (Stata Corporation, College Station, TX, USA), using two-sided P values.

## Results

### Eligible studies

Based on the search strategy, two studies were selected. The two trials enrolled 4107 breast cancer patients, of whom 2058 received a taxane–anthracycline–capecitabine-containing regimen and 2049 received a taxane–anthracycline-based regimen. All of the patients were histologically confirmed as having invasive breast cancer. These two studies had study population and trial design in common. The study details are shown in Table 1.

**Table 1:** Characteristics of studies included for the meta-analysis.

study	patients	treatment	Cycles	Duration of follow-up
	per treatment arm			
USO	1304	Epirubicin+Cyclophosphamide q3w	4	5 years
		Docetaxel q3w	4	
	1307	Epirubicin+Cyclophosphamide q3w	4	
		Docetaxel+Capecitabine q3w	4	
FinXX	745	Docetaxel q3w	3	5 years
		+Epirubicin+Cyclophosphamide+5- fluorouracil q3w	3	
	751	Docetaxel++Capecitabine q3w	3	
		+Epirubicin+Cyclophosphamide+5- fluorouracil+Capecitabine q3w	3	

### Meta-analysis of the primary endpoint

**Figure 1:**
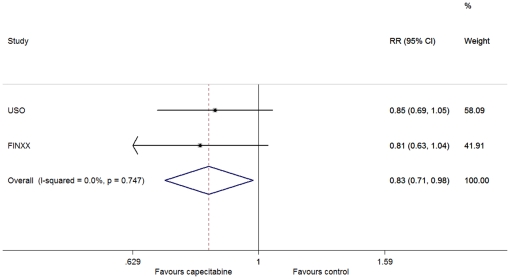
Forest plot of meta-analysis on the disease-free survival for the addition of capecitabine to standard treatment.

There was no between-study heterogeneity in DFS (heterogeneity χ^2^ = 0.10 (d.f. = 1), I^2^ = 0.0%, P = 0.747). Therefore, we used the fixed-effect model to analyze the data and found that DFS was significantly improved in the capecitabine arm versus the controls (HR = 0.83, 95% CI: 0.71–0.98; P = 0.027) (figure 1).

### Meta-analysis of the secondary endpoints

**Figure 2:**
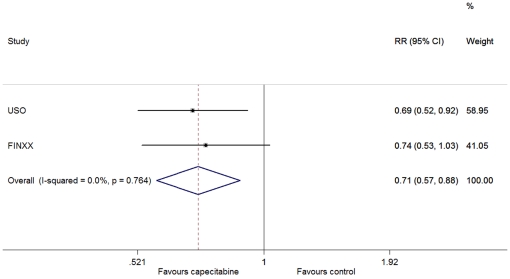
Forest plot of meta-analysis on the overall survival for the addition of capecitabine to standard treatment.

**Figure 3:**
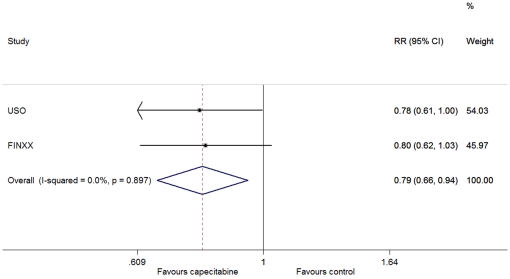
Forest plot of meta-analysis on the distant recurrence for the addition of capecitabine to standard treatment.

**Figure 4:**
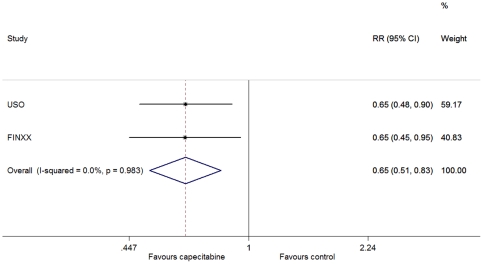
Forest plot of meta-analysis on the breast cancer specific survival for the addition of capecitabine to standard treatment.

There was no between-study heterogeneity in HRs of the studies (heterogeneity χ^2^ = 0.09 (d.f. = 1), I^2^ = 0.0%, P = 0.764) and the addition of capecitabine to standard treatment showed improvement in OS (HR = 0.71, 95% CI: 0.57–0.88; P = 0.002) (figure 2). For the distant recurrence, through the fixed-effect model (heterogeneity χ^2^ = 0.02 (d.f. = 1), I^2^ = 0.0%, P = 0.897), we also observed a significant improvement in favor of the capecitabine-based arm (HR = 0.79, 95% CI: 0.66–0.94; P = 0.008) (figure 3). We used the fixed-effect model (heterogeneity χ^2^ = 0·00 (d.f. = 1), I^2^ = 0.0%, P = 0.983) to analyze the breast-cancer-specific survival, and found that there was a significant difference between the two arms (HR = 0.65, 95% CI: 0.51–0.83, P = 0.001) (figure 4). However, no difference was discerned in death from other causes between patients with and without the addition of capecitabine (heterogeneity χ^2^ = 0.31 (d.f. = 1), I^2^ = 0.0%, P = 0.576; HR = 1.07, 95% CI: 0.63–1.82, P = 0.798).

### Subgroup analysis

**Figure 5:**
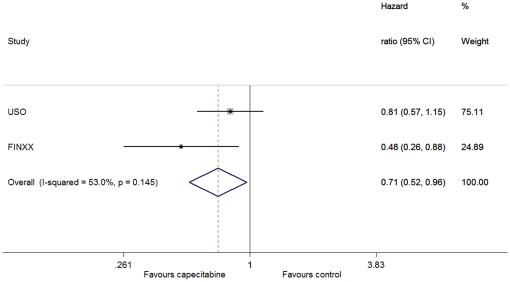
Forest plot of meta-analysis on the disease-free survival in triple negative patients for the addition of capecitabine to standard treatment.

**Figure 6:**
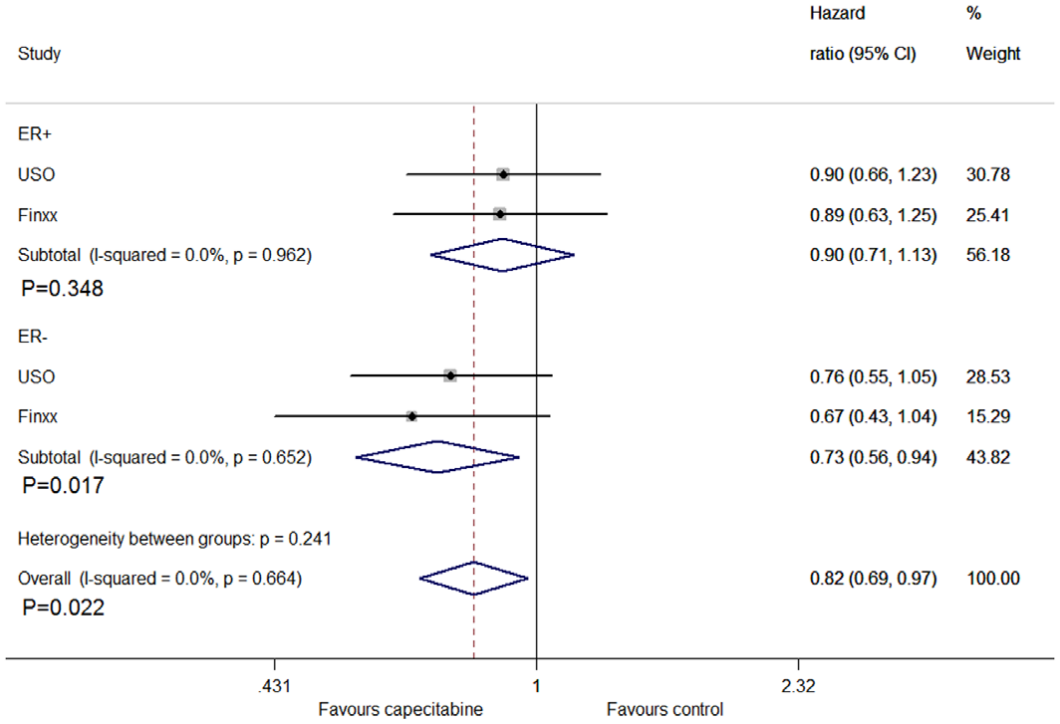
Forest plot of meta-analysis on the disease-free survival in hormone receptor positive and negative patients for the addition of capecitabine to standard treatment.

**Figure 7:**
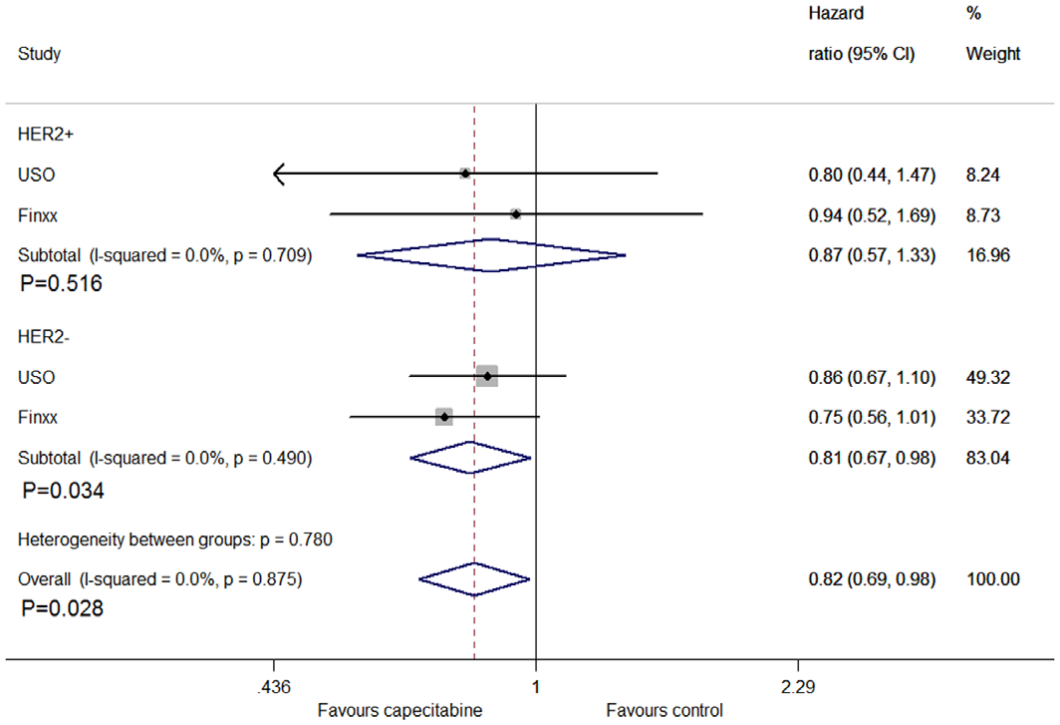
Forest plot of meta-analysis on the disease-free survival in HER2 positive and negative patients for the addition of capecitabine to standard treatment.

For the subgroup analysis, we divided them into triple negative breast cancer patients and non-triple negative breast cancer patients. From the analysis, we found that capecitabine improved DFS in triple negative patients (HR = 0.71, 95% CI: 0.53–0.96, P = 0.028), by fixed method (figure 5). For the different hormone receptor status, no significant difference was found between the groups in hormone receptor positive patients (HR = 0.90, CI: 0.71–1.13, P = 0.348), but we exactly found the difference between the groups in hormone receptor negative patients (HR = 0.73, CI: 0.56–0.94, P = 0.017) (figure 6).The association between DFS and HER2 status were not statistically significant in HER2 positive patients (HR = 0.87, CI: 0.57–1.33, P = 0.516), but revealed a significant difference in HER2 negative counterparts (HR = 0.81, CI: 0.67–0.98, P = 0.034) (figure 7).

### Publication bias and sensitivity analyses

We performed the funnel plots and Begger's test to assess the publication bias. As a result, there was no publication bias in each test (z = −1.00, P = 0.317) for the primary endpoint analysis and the secondary endpoint analysis (data not shown). The influence of individual studies on the summary effect estimate was performed by sensitivity analyses on the overall HR. No individual study affected the overall HR, because omission of any single study made no significant difference.

## Discussion

This meta-analysis aimed to assess the efficacy of addition of capecitabine to anthracycline–taxane-based adjuvant therapy in high-risk early breast cancer for the first time. First, it was reasonable to merge these two large clinical trials because of their similar regimens for control (anthracycline–taxane-based polychemotherapy) and experiment arm (anthracycline–taxane–capecitabine-based polychemotherapy). Second, capecitabine in combination with docetaxel is synergic and effective and is indicated for treatment of metastatic breast cancer [12]. Therefore, it may also improve prognosis of adjuvant therapy. The present meta-analysis does demonstrate the clinical benefits of addition of capecitabine to polychemotherapy.

Many trials, such as PACS01 and NSABP B28, have demonstrated that taxane-containing regimens improve DFS and OS in an adjuvant treatment for breast cancer patients [13]. Capecitabine is a prodrug that is enzymatically metabolized in the liver to fluorouracil, and it eventually inhibits DNA synthesis and function. On the other hand, its synergistic effect with docetaxel and other cytotoxic drugs can be attributed to an increase in TP level in the tumors [14], [15]. The control regimens of the USON 01062 (AC-T) and Finxx (T-CEF) trials may be similar in efficacy, although the efficacy of the T-CEF regimen is still under investigation. These two large trials failed to provide a satisfactory outcome, but from the current meta-analysis, we found that capecitabine plus standard chemotherapy significantly improved OS, DFS, local recurrence, distant recurrence, and breast-cancer-specific survival. Due to the synergistic effect of taxane and capecitabine, taxane–anthracylcine-based regimens with capecitabine may effectively inhibit distant micrometastases, to further improve the efficacy, and could be a novel combination chemotherapy regimen. A previous study showed that adjuvant capecitabine monotherapy is not superior to CMF administered every 3 weeks. However, combination regimens with capecitabine may provide benefits over single-agent therapy because the combined regimen enhances the control of metastatic foci [16].

Our meta-analysis provides the efficacy of capecitabine to some subtypes of early breast cancer patients, such as hormone receptor negative, HER2 negative and triple negative cancers. This was also showed in ABCSG-24 trial that addition of capecitabine to epirubicin plus docetaxel is associated with a greater chance of achieving pCR when the cancer is hormone receptor negative [17]. Confirmation of these findings require further research but interactions of capecitabine with other chemotherapy agents, especially in some biologic shugroups of early breast cancer. Triple negative patients have an absence of estrogen receptor, progesterone receptor and HER2/neu expression, and have worse prognosis [18], [19]. Furthermore, there is no standard adjuvant treatment for this category of patient [20]. Some trials have provided a clue for the striking effect of taxane-containing polychemotherapy in improving breast cancer in triple negative patients [21], [22], [23]. However, our meta-analysis suggests that addition of capecitabine to anthracycline–taxane-based polychemotherapy is better than anthracycline–taxane-based polychemotherapy, and therefore, might be one of the good regimens for adjuvant therapy of triple negative breast cancer.

Capecitabine is an oral, tumor-targeted drug. From previous trials, we have found fewer adverse reactions with docetaxel and capecitabine compared with anthracycline or paclitaxel [7], [24], [25]. To some extent, addition of capecitabine did not significantly increase the toxicity, except for diarrhea and hand–foot syndrome related to capecitabine, but it might have reduced neutropenia and febrile neutropenia, which could have been due to reduced dose of docetaxel [3].

There were some limitations to our meta-analysis. Only two trials were included in this analysis. Fortunately, all encouraging results were yielded in the ITT analysis, which provides powerful evidence to support capecitabine-based polychemotherapy as a good candidate regimen for adjuvant therapy of early breast cancer. We are looking forward for more clinical trials to perfect this hypothesis. However, the two trials were very similar, and therefore this meta-analysis might be highly persuasive.

Above all, the results of the current meta-analysis indicate that taxane-based regimens with capecitabine may be effective, convenient, and well-tolerated in patients with early breast cancer; especially in triple negative patients.
